# Human motor neurons derived from induced pluripotent stem cells are susceptible to SARS-CoV-2 infection

**DOI:** 10.3389/fncel.2023.1285836

**Published:** 2023-12-05

**Authors:** Gioia Cappelletti, Claudia Colombrita, Fiona Limanaqi, Sabrina Invernizzi, Micaela Garziano, Claudia Vanetti, Claudia Moscheni, Serena Santangelo, Silvia Zecchini, Daria Trabattoni, Vincenzo Silani, Mario Clerici, Antonia Ratti, Mara Biasin

**Affiliations:** ^1^Laboratory of Immune-Biology, Department of Biomedical and Clinical Sciences, University of Milan, Milan, Italy; ^2^Department of Neurology and Laboratory of Neuroscience, IRCCS Istituto Auxologico Italiano, Milan, Italy; ^3^Laboratory of Immunology, Department of Pathophysiology and Transplantation, University of Milan, Milan, Italy; ^4^Department of Medical Biotechnology and Translational Medicine, Aldo Ravelli Center for Neurotechnology and Experimental Brain Therapeutics, University of Milan, Milan, Italy; ^5^Department of Pathophysiology and Transplantation, “Dino Ferrari” Center, University of Milan, Milan, Italy; ^6^Don C. Gnocchi Foundation, Istituto di Ricovero e Cura a Carattere Scientifico (IRCCS) Foundation, Milan, Italy

**Keywords:** SARS-CoV-2 infection, iPSC-derived motor neurons, long-COVID, neuroinflammation, neuromuscular disorders, COVID-19

## Abstract

**Introduction:**

COVID-19 typically causes Q7 respiratory disorders, but a high proportion of patients also reports neurological and neuromuscular symptoms during and after SARSCoV-2 infection. Despite a number of studies documenting SARS-CoV-2 infection of various neuronal cell populations, the impact of SARS-CoV-2 exposure on motor neuronal cells specifically has not been investigated so far.

**Methods:**

Thus, by using human iPSC-derived motor neurons (iPSC-MNs) we assessed: (i) the expression of SARS-CoV-2 main receptors; (ii) iPSC-MN infectability by SARS-CoV-2; and (iii) the effect of SARS-CoV-2 exposure on iPSC-MN transcriptome.

**Results:**

Gene expression profiling and immunofluorescence (IF) analysis of the main host cell receptors recognized by SARS-CoV-2 revealed that all of them are expressed in iPSC-MNs, with CD147 and NRP1 being the most represented ones. By analyzing SARS-CoV-2 N1 and N2 gene expression over time, we observed that human iPSC-MNs were productively infected by SARS-CoV-2 in the absence of cytopathic effect. Supernatants collected from SARS-CoV-2-infected iPSC-MNs were able to re-infect VeroE6 cells. Image analyses of SARS-CoV-2 nucleocapsid proteins by IF confirmed iPSC-MN infectability. Furthermore, SARS-CoV-2 infection in iPSCMNs significantly altered the expression of genes (IL-6, ANG, S1PR1, BCL2, BAX, Casp8, HLA-A, ERAP1, CD147, MX1) associated with cell survival and metabolism, as well as antiviral and inflammatory response.

**Discussion:**

These results suggest for the very first time that SARS-CoV-2 can productively infect human iPSC-derived MNs probably by binding CD147 and NRP1 receptors. Such information will be important to unveil the biological bases of neuromuscular disorders characterizing SARS-CoV-2 infection and the so called long-COVID symptoms.

## Introduction

SARS-CoV-2 infection typically causes respiratory disorders, but a surprisingly high proportion of patients also reports neurological and neuromuscular complications during and after the acute phase of Corona Virus Disease 19 (COVID-19) (Koralnik and Tyler, 2020; Mao et al., 2020; Chou et al., 2021; Nalbandian et al., 2021). These include cerebrovascular disease, seizures, meningitis, encephalitis, loss of smell (anosmia), taste (ageusia) and myositis (Pezzini and Padovani, 2020; Klein et al., 2021). Moreover, even several months after recovery, almost 30% of the patients display prolonged symptoms including deficits of memory and attention, insomnia, anxiety, depression, a dysexecutive syndrome consisting of inattention, disorientation, and poor movement coordination (Jaywant et al., 2021; Méndez et al., 2021; Ferrucci et al., 2022), and also ataxia, muscle aches and joint pains (Davis et al., 2021; Huang et al., 2021; Tomasoni et al., 2021).

Besides the Central Nervous System (CNS), the documented symptoms also imply an alteration of the peripheral nervous system (PNS) following SARS-CoV-2 infection, which could trigger or worsen neurodegenerative disorders, as recently reported by Serrano-Castro et al. (2020). Supporting this hypothesis, peripheral nerve damage and a more generalized acute polyneuropathy, known as the Guillain-Barré syndrome (GBS) (Needham et al., 2020; Pezzini and Padovani, 2020; Abu-Rumeileh et al., 2021; Raahimi et al., 2021; Taguchi et al., 2022; Stępień and Pastuszak, 2023), have been reported in SARS-CoV-2-infected patients. In three COVID-19 patients, a possible association with a new diagnosis of myasthenia gravis was documented (Restivo et al., 2020; Muhammed et al., 2021). Furthermore, peripheral motor neuropathy has been described before the onset of the typical flu-like symptoms of COVID-19 (Caress et al., 2020; Zhao et al., 2020) and diagnostic criteria for acute polyradiculoneuropathy have been described as well (Alberti et al., 2020). Further supporting the detrimental effect of coronaviruses on PNS, both GBS and acute motor axonal neuropathy (AMAN) have been associated to SARS and MERS infections (Kim et al., 2017; Andalib et al., 2021).

The aforementioned sequelae contribute to defining a clinical picture commonly referred to as “Long COVID-19” or “Post COVID-19” (Carfì et al., 2020; Garrigues et al., 2020),^1^ which might also occur as a consequence of direct infection/exposure of neuronal cells to SARS-CoV-2 (Lyons-Weiler, 2020). Indeed, *ex vivo*, *in vivo*, and *in vitro* studies suggest that SARS-CoV-2 is able to infect different kinds of neuronal populations, with different degrees of success (Pellegrini et al., 2020; Ramani et al., 2020; Zhang et al., 2020; Song et al., 2021; Lopez et al., 2022; Lyoo et al., 2022; Stein et al., 2022; Kettunen et al., 2023). Our understanding of the mechanisms through which SARS-CoV-2 can enter the nervous system and infect nerve cells is still not completely clear. Studies from both post-mortem COVID-19 patients and animal models suggest that SARS-CoV-2 exploits different neuroinvasive strategies and accession routes (Nampoothiri et al., 2020; Zhou et al., 2020; Anand et al., 2021). These include: (i) hematogenous transport of infected immune cells via the circulatory system of the brain tissue; (ii) infection of the nasal olfactory epithelium to reach the brain by axonal transport along the olfactory nerve; (iii) retrograde virus spread from the lungs to the CNS through the vagus nerve; (iv) entry from the ocular epithelium; and (v) virus invasion through impairment of the blood-brain barrier (BBB).

The molecular mechanisms sustaining SARS-CoV-2 infection of nerve cells are yet to be defined as well. For instance, angiotensin converting enzyme 2 (ACE2), the major actor involved in multi-organ SARS-CoV-2 infection, shows low expression levels in the human brain (Hamming et al., 2004). Thus, despite the initial concept of ACE2 distribution as the major determinant of SARS-CoV-2 infectivity and spread, other factors have been called-on as responsible for SARS-CoV-2 neurovirulence. This is the case of Neuropilin 1 (NRP1) (Cantuti-Castelvetri et al., 2020; Daly et al., 2020), CD147 (BSG) (Chen et al., 2020), transmembrane serine protease 2 (TMPRSS2) (Qiao et al., 2020), and Furin (Coutard et al., 2020; Ou et al., 2020), which show higher and broader patterns of expression in neuronal cells compared to ACE2.^2^

**FIGURE 1:**
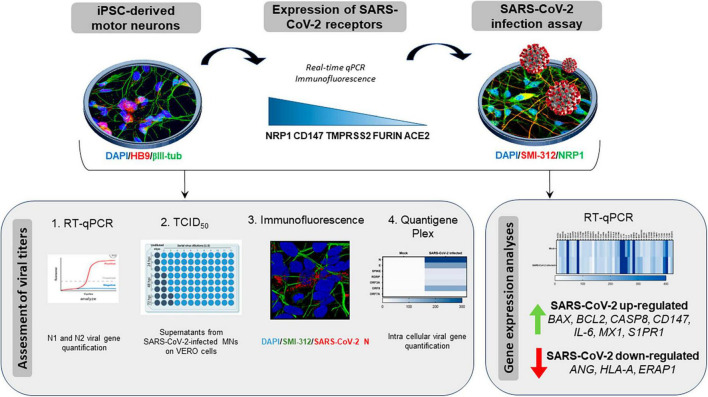
Graphical representation of the study workflow. SARS-CoV-2 host receptor gene expression was assessed on iPSC-derived motor neurons (iPSC-MNs) by RT-qPCR and immunofluorescence. iPSC-MN were then *in vitro* infected by SARS-CoV-2 and viral infection/replication was assessed by a multidisciplinary approach using RT-qPCR, TCID_50_, immunofluorescence and quantigene Plex techniques. The effect of viral infection on iPSC-MN homeostasis was determined by analyzing the alteration of their transcriptome.

Although public health measures and vaccination campaigns have significantly contributed to limiting COVID-19 spread and severity, SARS-CoV-2-induced neurological manifestations still represent a threat for long-term health, as their biological bases remain largely unknown. In particular, the documented neuromuscular dysfunctions related with COVID-19 suggest the occurrence of widespread alterations possibly affecting all the motor unit components. Nonetheless, the impact of SARS-CoV-2 exposure on motor neuronal cells specifically has not been investigated so far. Therefore, as summarized in Figure 1, by using an *in vitro* model of human motor neurons differentiated from induced pluripotent stem cells (iPSC-MNs), we aimed to assess, for the first time (i) the expression of SARS-CoV-2 main receptors; (ii) iPSC-MN infectability by SARS-CoV-2; and (iii) the effect of SARS-CoV-2 exposure on iPSC-MN transcriptome.

## Materials and methods

### Cell lines and culture

VeroE6 cells (ATCC, VA, USA) and Human Lung Carcinoma Cells Expressing Human Angiotensin-Converting Enzyme 2 (A549-hACE2) (BEI Resources, Catalog No. NR-53821) were grown in Dulbecco’s Modified Eagle’s Medium (DMEM) (Euroclone, Milan, Italy) containing 4 mM L-glutamine, supplemented with 10% FBS, 100 μ/mL penicillin and 100 μg/mL streptomycin at 37°C and 5% CO_2_. Cells were regularly passaged and tested for the presence of mycoplasma contamination.

### iPSC generation and MN differentiation

Induced Pluripotent Stem Cells (iPSC) were reprogrammed from 3 healthy donors (Supplementary Table 1) after obtaining informed consent and approval from local ethics committee (approval number 2022_03_15_12). For fibroblast/blood cell reprogramming the CytoTune^®^-iPSC 2.0 Sendai Reprogramming Kit (Thermo Fisher Scientific) was used as previously described (Bardelli et al., 2020). After picking and selecting about 6 clones per sample, one clone for each cell line was further grown in Essential 8 medium (Thermo Fisher Scientific) and fully characterized for the expression of stemness markers (TRA-1-60, OCT3/4, SOX2, NANOG) and for the capacity to spontaneously differentiate into the three germ layers lineages as assessed by the expression of alpha-feto protein (AFP) as endodermal, βIII tubulin as ectodermal and desmin as mesodermal markers and already shown in Bossolasco et al. (2018) and Gumina et al. (2019). Genome integrity of the iPSC clones was determined by Q-banding karyotype analysis (Bossolasco et al., 2018).

Induced Pluripotent Stem Cells were differentiated into motoneurons (MNs) as previously described (Bardelli et al., 2020). Briefly, iPSCs were grown in suspension for 21 days to obtain embryoid bodies (EBs) in HuES medium (DMEM/F12, 20% knock-out serum replacement, 2 mM L-glutamine, 10 μ/ml penicillin, 10 μg/ml streptomycin, 0.1 mM MEM NEAA, 110 μM β-mercaptoethanol) for the first 3 days and then in neural induction medium (DMEM/F12, 2 mM L-glutamine, 10 μ/ml penicillin, 10 μg/ml streptomycin, 0.1 mM MEM NEAA, 2 μg/ml heparin, 1% N2 supplement), supplemented with specific factors. EBs were dissociated with 0.05% trypsin and cells were plated on poly-D-lysine/laminin-coated (Thermo Fisher Scientific) coverslips and cultured in neural differentiation medium (Neurobasal medium, 2 mM L-glutamine, 10 μ/ml penicillin, 10 μg/ml streptomycin, 0.1 mM MEM NEAA, 1% N2 supplement, all from Thermo Fisher Scientific) with the addition of specific factors for 13 days to obtain iPSC-MNs.

### *In vitro* SARS-CoV-2 infection assay

The European (EU–B.1) SARS-CoV-2 lineage was a kind gift of Dr. Davide Mileto, Clinical Microbiology, ASST Fatebenefratelli-Sacco, Milan, Italy. All the experiments with SARS-CoV-2 were performed in a BSL3 facility.

In order to generate a viral stock, SARS-CoV-2 was expanded in VeroE6 cells and infectious viral particles concentration was assessed by 50% tissue culture infectious dose (TCID_50_) assay, as elsewhere described (Fenizia et al., 2022).

Induced Pluripotent Stem Cells-MNs were *in vitro* Mock- or SARS-CoV-2-infected with 1 multiplicity of infection (MOI). After an overnight incubation, cells were thoroughly washed three times with pre-warmed PBS and replenished with the complete neural differentiation medium. Supernatants were collected at 6 (T0), 24 (T1), 48 (T2), and 72 (T3) hours post-infection (hpi) to monitor viral replication and to perform SARS-CoV-2 infection assays on VeroE6 cells for each iPSC-MN line (Supplementary Table 1 and Supplementary Figure 1).

At 48 hpi, iPSC-MNs were fixed for immunofluorescence (IF) analyses, while cells harvested at 72 hpi were lysed for RNA extraction and appropriately stored at −80°C for further processing, as specified below.

At 72 hpi, cell viability was assessed by Trypan Blue exclusion assay. Briefly, iPSC-MNs were incubated in Accutase (Thermo Fisher Scientific) for 5 min at 37°C. Then, an equal volume of fresh medium was added to the wells to stop the dissociation reaction and the cells were detached and centrifuged for 8 min at 1200 rpm. The supernatant was carefully discarded, and cells were resuspended in 1 mL of fresh medium. Ten μl of cell suspension were incubated with 10 μl of 0.4% Trypan Blue (Merck-Sigma, Milan, Italy) in 96-well plates. Ten μl of the mix were loaded on chamber slides and counted with the T20 Automated Cell Counter (Bio-Rad Laboratories, Hercules, CA, USA).

### Viral replication assessment

For SARS-CoV-2 replication assessment, RNA was extracted from iPSC-MN supernatants using the Maxwell^®^ RSC Instrument with Maxwell^®^ RSC Viral Total Nucleic Acid Purification Kit (Promega, Fitchburg, WI, USA). Viral RNA was reverse transcribed in a single-step RT-qPCR (GoTaq 1-Step RT-qPCR; Promega) on a CFX96 instrument (Bio-Rad, Hercules, CA, USA) using primers specifically designed to target two regions of the nucleocapsid (N1 and N2) gene (Fenizia et al., 2020) (2019-nCoV CDC qPCR Probe Assay emergency kit; IDT, Coralville, IA, USA), together with primers for the human RNase P gene. Viral copy number quantification (viral copy number/μl) was assessed by creating a standard curve from the quantified 2019-nCoV_N positive Plasmid Control (IDT).

VeroE6 cells were *in vitro* infected with supernatants collected from SARS-CoV-2-infected iPSC-MNs at different time points (24, 48, 72 hpi) and infectious viral particles concentration was assessed by TCID_50_, as previously described. Briefly, VeroE6 cells were seeded at 2 × 10^4^ cells per well in a 96-well plate and cultured with serial dilutions (1:3) of the iPSC-MN supernatants collected at different time points. After 72 hpi, VeroE6 cell supernatants were removed, cells fixed by 4% paraformaldehyde (PFA–Sigma-Aldrich, MO, USA) for 1 h at RT and then stained with 0.2% crystal violet solution (Sigma-Aldrich) to assess cell death and to calculate TCID_50_.

### QuantiGene plex gene expression assay

SARS-CoV-2 infection was further assessed on 5 × 10^4^ iPSC-MNs by QuantiGene Plex assay (Thermo Scientific), which uses signal amplification rather than target amplification for direct measurement of RNA transcripts directly from lysed cells. The following SARS-CoV-2 viral genes were analyzed: *ORF7A*, *ORF3A*, *ORF8*, *RDRP*, *E*, and *N*. Signal was detected using a Luminex instrument and results were calculated relative to GAPDH, and PPIB as housekeeping genes, and expressed as ΔCt.

### Gene expression analyses

Total RNA was extracted from iPSC-MNs as previously described by Limanaqi et al. (2022). Gene expression analyses of the main SARS-CoV-2 receptors (ACE2, CD147, NRP1) and peptidases (FURIN, TMPRSS2) as well as N (nucleocapsid)1, N2, S (spike)1, S2 and E (envelope)1 SARS-CoV-2 sequences was performed by Real-time qPCR (CFX96 connect, Bio-Rad, Hercules, CA, USA) using SYBR Green PCR mix (Promega), according to the following thermal profile: initial denaturation (95°C, 15 min), and 40 cycles with denaturation (15 s at 95°C), annealing (1 min at 60°C) and extension (20 s at 72°C). A Ct value of 35 or higher was considered negative. Melting curves were also analyzed for amplicon characterization. Results for gene expression analyses were calculated by the 2^–ΔΔCt^ equation and presented as the average of the relative expression units to an internal reference sample and normalized to the expression of the GAPDH housekeeping gene. Samples with GAPDH Ct values above 20 were excluded from the analysis. Already optimized primers were purchased (PrimePCR, Bio-Rad, Segrate, Italy). Gene expression analyses of the main SARS-CoV-2 receptors was also assessed on RNA extracted from A549-hACE2 cells, as positive control.

The expression of 46 genes related to inflammatory, apoptotic, and antiviral pathways were analyzed by a PCR array including a set of optimized Real-time PCR primers (Bio-Rad) for the targets reported in Supplementary Table 2. Gene expression analyses were performed in iPSC-MNs at 72 hpi in duplicates. Results were analyzed using the SABiosciences online software, expressed by the 2^–ΔΔCt^ equation and presented as the average of the relative expression units to an internal reference sample and normalized to the expression of the GAPDH and ACTB housekeeping genes.

### Immunofluorescence assays

Induced Pluripotent Stem Cells-MNs were seeded on coverslips in a 24-well plate, cultured until differentiation, and infected as specified above. At 48 hpi, cells were fixed in PBS containing 4% PFA at RT for 10 min, followed by permeabilization with 0,1% TritonX-100 in PBS for 10 min. Cells were treated with 1% BSA in PBS for blocking at RT for 1 h, and incubated at 4°C overnight with specific primary antibodies. The following primary antibodies were used: anti-beta III Tubulin (1:500, Abcam, Cambridge, UK), anti-SMI-312 (1:1000, Covance, Princetown, NJ, USA) and anti-ChAT (1:200, Chemicon) to assess iPSC-MN differentiation; anti-ACE2 (1:200, Prodotti Gianni), anti-CD147 (1:100, Thermo Fisher Scientific) anti-NRP1 (1:100, Thermo Fisher Scientific) and anti-N Nucleocapsid SARS-CoV-2 (1:1000, BEI Resources) to assess SARS-CoV-2 receptors and infection. Coverslips were then stained with secondary antibodies (Alexa Fluor 488 or 647, 1:500, Abcam) for 45 min at RT and mounted using a medium containing DAPI (Enzo Life Sciences, Milan, Italy). Confocal images were acquired on a TCS SP8 System equipped with a DMi8 inverted microscope and a HC PL APO 40 × /1.30 Oil CS2 (Leica Microsystems, Wetzlar, Germany) at a resolution of 1024 × 1024 pixels (single stack).

### Statistical analyses

Overall, we performed 14 SARS-CoV-2 independent experiments by using iPSC-derived MNs from 3 healthy donors. The different analyses were assessed on such samples according to the scheme reported in Supplementary Table 1.

Statistical analyses were performed using GraphPad Prism 8. Results are expressed as mean ± SEM of the indicated *n*-values. The two-tailed Student’s *t*-test was used with a *p*-value threshold of 0.05.

## Results

### Expression of SARS-CoV-2 receptors in iPSC-derived MNs

**FIGURE 2:**
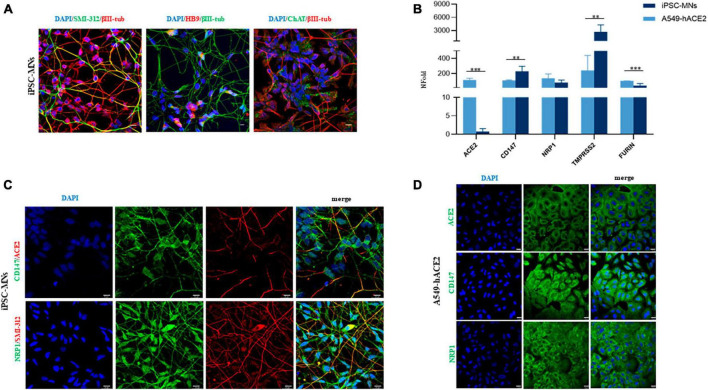
Expression of SARS-CoV-2 human receptors on iPSC-MNs and A549-hACE2 cells. **(A)** Representative images of differentiated iPSC-MNs obtained from 3 healthy control individuals. Expression of neuronal (βIII-Tubulin, red and green, and SMI-312, green) and motoneuronal (HB9, red and ChAT, green) markers is shown in merged images. Nuclei were stained with DAPI (blue). Bar, 10 μm. **(B)** Gene expression analyses of ACE2, CD147, NRP1, TMPRSS2, and FURIN in A549-hACE2 cells and iPSC- MNs by Real-time qPCR. Results are presented as mean ± SEM; *n* ≥ 4 for each cell line/iPSC-MN; the Student’s *t*-test was used with the *p*-value threshold of 0.05. Significance is indicated as follows: **p < 0.01; ***p < 0.001. Representative immunofluorescence images for CD147 and NRP1 markers (green) in iPSC-MNs and in A549-hACE2 cells are shown in panels **(C,D)**, respectively. The expression of ACE2 is shown in red **(C)** and in green **(D)**. Nuclei were stained with DAPI (blue). The neuronal marker SMI-312 (red) is shown only in **(C)**. Bars correspond to 20 μm in both **(C,D)**.

To assess the potential susceptibility of motor neuronal cells to SARS-CoV-2 infection, we differentiated iPSC from 3 healthy donor individuals (1 male and 2 females, 37–49 years of age at biopsy collection; Supplementary Table 1) into motor neurons (iPSC-MNs) expressing neuronal (bIII-tubulin and SMI-312) and motoneuronal (ChAT, HB9) markers (Figure 2A). By Real-time qPCR we validated the gene expression of the main receptors used by the virus and observed that all SARS-CoV-2 receptors (ACE2, CD147, NRP1) and peptidases (TMPRSS2, FURIN) analyzed were expressed in iPSC-MNs, although with different degrees (Figure 2B). In particular, by assessing the expression of these receptors in A549-hACE2 cells, a cell line used as positive control, ACE2 and Furin gene expression was significantly lower in iPSC-MNs compared to A549-hACE2 cells (Figure 2B). NRP1 gene expression was instead comparable in iPSC-MNs and A549-hACE2 cells, while CD147 and TMPRSS2 gene expression was significantly higher in iPSC-MNs than in A549-hACE2 cells (Figure 2B). Gene expression data on SARS-CoV-2 human receptors were further confirmed by IF analysis for ACE2, CD147 and NRP1 markers on both iPSC-MNs (Figure 2C) and A549-hAC2 cells (Figure 2D). Indeed, as for the gene expression analysis, ACE2 fluorescence intensity was significantly lower in iPSC-MNs compared to A549-hACE2 cells (Supplementary Figure 1).

### SARS-CoV-2 viral replication in iPSC-MNs

To assess whether iPSC-MNs are productively infected by SARS-CoV-2, different experimental approaches were employed. We first assessed that cell viability, measured by Trypan blue assay, was not significantly modified in the 3 different iPSC-MN lines by comparing mock- and SARS-CoV-2-infected cells which showed more than 90% viability (Supplementary Table 3), indicating a lack of cytopathic effect.

**FIGURE 3:**
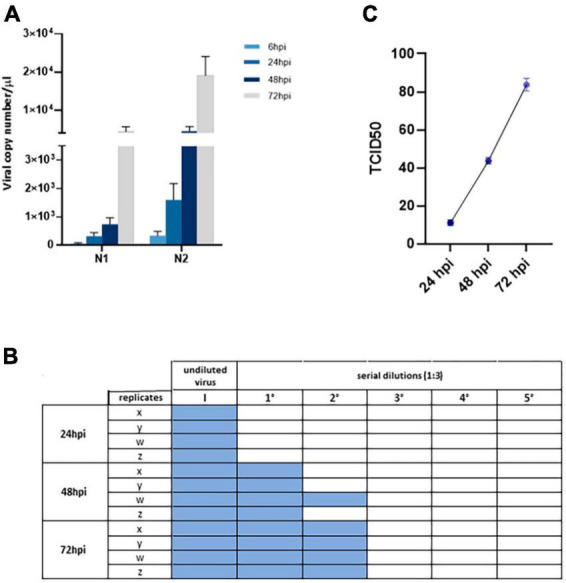
Assessment of viral replication in iPSC-MNs. **(A)** Upon *in vitro* challenge of iPSC-MNs with 1 MOI of SARS-CoV-2, the infection was monitored at 6, 24, 48, and 72 hpi. Results correspond to the absolute viral copy number/μl of the SARS-CoV-2 N1 and N2 target sequences from cell supernatants that were quantified through a single-step Real-time qPCR by referring to a standard curve for Ct values (IDT, Coralville, IA, USA). Results are presented as mean ± SEM from ≥4 independent replicates each on iPSC-MNs derived from the 3 enrolled healthy subjects (Supplementary Table 1). **(B)** TCID_50_ analyses on VeroE6 cell infectability after exposure to iPSC-MN-infected supernatants, specifically the undiluted virus (I) and five serial dilutions (1:3), at 24, 48, and 72 hpi. Viral infection was assessed by cytopathic effect on VeroE6 cells as represented by colored well. The plate is representative of a single experiment which was performed once in quadruplicate (x, y, z, w) for each of the three different iPSC-MN lines. **(C)** Titration of SARS-CoV-2 virus in VeroE6 supernatants at 24, 48, and 72 hpi from data shown in **(B)**. Data are shown as TCID_50_. Results are presented as mean ± SEM from four independent replicates each on iPSC-MNs derived from the three enrolled healthy subjects.

By analyzing N1 and N2 viral nucleocapsid gene expression by Real-time qPCR in supernatants from iPSC-MN cultures over a timeframe of 72 hpi, we observed that human iPSC-MNs were productively infected by SARS-CoV-2 in a time-dependent manner (Figure 3A), although viral replication was not accompanied by cytopathic effect as assessed by crystal violet assay (data not shown). Moreover, levels of viral replication were modest (at 72 hpi: mean viral copy number/μl ± SEM, N1 = 4464.2 ± 1281.7; N2 = 19139.4 ± 5157.0) compared with SARS-CoV-2-susceptible VeroE6 cells (at 72 hpi: mean viral copy number/μl ± SEM, N1 = 18.18e + 06 ± 3.2 e + 06; N2 = 80.93e + 06 ± 17.29 e + 06) (Supplementary Figure 2).

To further verify the productive infectability of iPSC-MNs, we then exposed VeroE6 cells to supernatants collected from infected iPSC-MNs at different time points (24, 48, and 72 hpi). VeroE6 cell infection resulted in an evident cytopathic effect which, as expected, increased according to the supernatant collection period over time (Figure 3B), from 9.5 TCID_50_ at 24 hpi to 85.4 TCID_50_ at 72 hpi (Figure 3C), mirroring the results obtained by Real-time qPCR.

**FIGURE 4:**
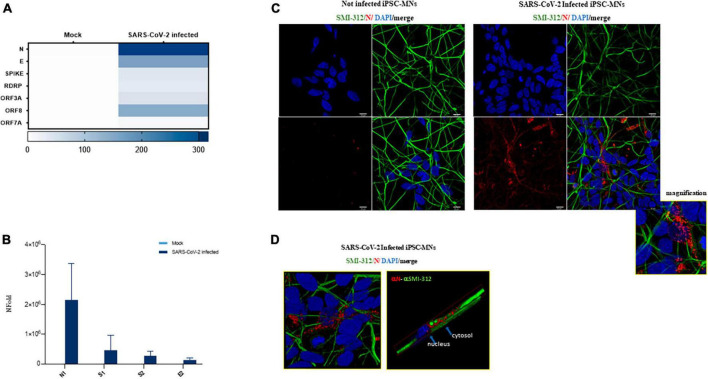
Assessment of viral replication in iPSC-MNs. **(A)** Expression of SARS-CoV-2 viral genes ORF7A, ORF3A, ORF8, RDRP, S, E, and N by QuantiGene Plex Gene expression technology in uninfected (Mock) and SARS-CoV-2-infected iPSC-MNs. Results shown on the heatmap correspond to the mean ± SEM from ≥1 independent replicates on each iPSC-MNs derived from 2 healthy control subjects (Supplementary Table 1). **(B)** Real-time qPCR expression analyses of N1, S1, S2, and E2 viral genes in uninfected (Mock) and SARS-CoV-2-infected iPSC-MNs. Results are presented as mean ± SEM from ≥4 independent replicates on iPSC-MNs derived from the 3 healthy control subjects (Supplementary Table 1). **(C)** Representative immunofluorescence images of N protein (red) and neuronal SMI-312 marker (green) in Mock- and SARS-CoV-2-infected iPSC-MNs at 48 hpi. Nuclei were stained with DAPI (blue). Bars correspond to 20 μm. **(D)** Representative magnified immunofluorescence images and 3D reconstruction for N protein and SMI-312 marker in SARS-CoV-2-infected iPSC-MNs at 48 hpi.

To further validate these results, the expression of some viral RNA targets was investigated in iPSC-MNs at intracellular level by three different methods: QuantiGene assay, Real-time qPCR and immunofluorescence (IF). As reported in the heatmap, the RNA of all the viral targets analyzed by QuantiGene (N, E, SPIKE, RDRP, ORF3A, ORF8, and ORF7A) were exclusively detected in SARS-CoV-2-infected cells, although at different levels (Figure 4A). The mRNA expression of N1, S1, S2, and E2 in infected iPSC-MNs was further confirmed by Real-time qPCR (Figure 4B).

Finally, by IF assay, the nucleocapsid (N) protein was detected exclusively in SARS-CoV-2-infected iPSC-MNs (Figure 4C), mainly at perinuclear level in the soma and along the neurite extensions (Figure 4D), though the percentage of infected cells seems to be very low.

### Effect of SARS-CoV-2 infection on iPSC-MN gene expression

**FIGURE 5:**
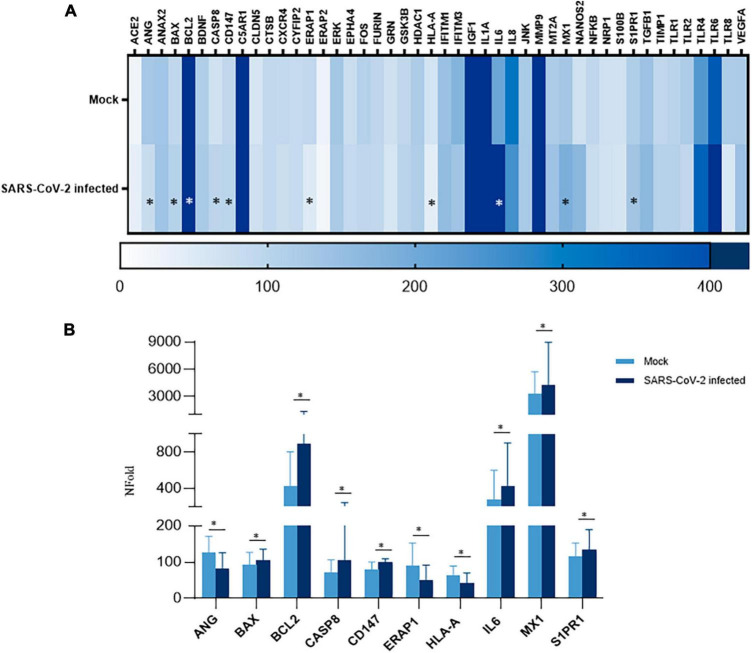
Gene expression analyses in SARS-CoV-2-infected iPSC-MNs. **(A)** Heatmap representation of Real-time qPCR expression data of 46 genes in uninfected (Mock) and SARS-CoV-2-infected iPSC-MNs at 72 hpi. **(B)**. Expression analyses of the statistically significant genes in SARS-CoV-2-infected vs. uninfected (Mock) iPSC-MNs at 72 hpi are shown in **(A)**. mRNA quantification was performed by Real-time qPCR and calculated by the 2–ΔΔCt equation. Results in **(A,B)** correspond to the mean ± SEM from ≥4 independent replicates on each iPSC-MN derived from the three enrolled healthy subjects (Supplementary Table 1); the Student’s *t*-test was used with the *p*-value threshold of 0.05. Significance is indicated as follows: *p < 0.05.

In order to assess whether SARS-CoV-2 infection fosters changes in iPSC-MNs gene expression, we evaluated the expression profile of a set of genes involved in the antiviral and immune-related response. Overall, among the 46 analyzed targets by a custom array (Supplementary Table 2), we observed a widespread alteration of gene expression following SARS-CoV-2 exposure at 72 hpi, suggesting a deep alteration of cell homeostasis (Figure 5A). Notably, SARS-CoV-2-induced deregulation was significant for 10 genes involved in different intracellular pathways with both up-regulated (BAX, BCL2, CASP8, CD147, IL-6, MX1, S1PR1) and down-regulated (ANG, HLA-A, ERAP1) expression, as shown in Figure 5B. Notably, despite a slight rise in CASP8 expression, the BCL2/BAX ratio was significantly increased in SARS-CoV-2 infected iPSC-MNs insinuating different plausible speculations about the effect triggered by viral entry on the apoptotic pathway (Supplementary Figure 3).

## Discussion

Clinical observations from COVID-19 patients support evidence for the damages caused by SARS-CoV-2 infection on both central and peripheral components of the NS. Documented neurological abnormalities include postural tremor (13.8%), motor/sensory deficits (7.6%) (Pilotto et al., 2021), as well as persistent muscle pain (myalgia), muscle weakness (mild to severe), fatigue, exercise intolerance and arthralgia (Carfì et al., 2020; Pleguezuelos et al., 2021; Soares et al., 2022). These are more frequent in severe manifestations of the disease and can differ among individuals (Montalvan et al., 2020; Niazkar et al., 2020). Rarely, clinically defined cases of acute disseminated encephalomyelitis, Guillain-Barré syndrome, and acute necrotizing encephalopathy have also been reported in COVID-19 patients (Lou et al., 2021). Furthermore, COVID-19 has been shown to significantly affect amyotrophic lateral sclerosis (ALS) patients, causing a rapid neurological deterioration, accompanied by a marked decline in fine motor skills of hand and leg strength (Fu et al., 2022). The damage of motor neurons, in turn, leads to the deterioration of muscle function, resulting in physical weakness, muscle atrophy, and paralysis. In addition, as reported by Li et al. (2022), SARS-CoV-2 infection may stimulate the ALS-associated amyloid aggregation of host proteins, providing molecular evidence for the role of SARS-CoV-2 in triggering neurodegeneration and MNDs.

Despite a number of studies documenting SARS-CoV-2 infection of various neuronal cell populations (Pellegrini et al., 2020; Ramani et al., 2020; Zhang et al., 2020; Lopez et al., 2022; Stein et al., 2022), to date, neither MN susceptibility to SARS-CoV-2 infection nor the molecular consequences of viral exposure on their homeostasis have been investigated. Therefore, it is still debated if the documented neurological/neuromuscular manifestations are secondary to a direct MN viral invasion and/or a collateral injury driven by an uncontrolled innate immune response fostering a pro-inflammatory milieu (immune mediated cytokines release), which is a hallmark of SARS-CoV-2 infection and COVID-19 severity.

In this study, we demonstrated for the first time that human iPSC-MNs are permissive to SARS-CoV-2 entry and production of infectious viral particles which are released in the cell culture supernatant. In fact, by using different experimental approaches, we verified that the virus is able to infect and replicate within this neuronal cell model. However, the levels of viral replication and the percentage of infected cells are significantly lower compared to those of susceptible cells, such as VeroE6 ones, possibly justifying the absence of cytopathic effect in iPSC-MNs. However, one should consider that this cellular model lacks the immunological component which, following SARS-CoV-2 infection, may favor the onset of a pro-inflammatory environment that is advantageous for viral infection/replication, and subsequent neuronal damage. It is therefore possible that in a pro-inflammatory setting, (i.e., in patients with pre-existing neuromuscular conditions) the rate of viral entry and replication would be substantially higher.

By profiling the expression of the main host cell receptors exploited by SARS-CoV-2, we confirmed previous data that ACE2 is scarcely expressed in neurons (Piras et al., 2022), here extending the observation to motor neuronal cells. We therefore speculate that iPSC-MN infection mainly relies on CD147 and/or NRP1 binding, as their expression on iPSC-MNs is high. Further confirming this assumption, it has already been demonstrated that these proteins have higher and broader patterns of expression in the human brain (Qiao et al., 2020), advocating for their role as putative mediators of SARS-CoV-2 entry into human nerve cells. However, further analyses will be necessary to validate this hypothesis.

Interestingly, we also observed that SARS-CoV-2 infection promotes an alteration of iPSC-MNs transcriptome, involving different intracellular pathways. First, SARS-CoV-2 infection was associated with upregulation of MX1, a protective factor whose expression was reported to be switched-on in several viral infections including SARS-CoV-2 (Sironi et al., 2014; Haller et al., 2018; Bizzotto et al., 2020; Haller and Kochs, 2020). This suggests that SARS-CoV-2 prompts the activation of the antiviral response in iPSC-MNs.

Second, in SARS-CoV-2-infected iPSC-MNs we observed a conspicuous transcriptional upregulation of the pro-inflammatory cytokine IL-6. Remarkably, significantly elevated IL-6 levels have been reported in COVID-19 patients, which is associated with adverse clinical outcome (Coomes and Haghbayan, 2020). In the NS, IL-6 levels increase in case of injury and/or inflammation, which may have both beneficial and detrimental effects on nerve cell survival and healing, depending on the cell type and context (Kummer et al., 2021). In the frame of neurodegenerative or neuropathic disorders, IL-6 exacerbates neurodegeneration and cell death, while blockade of IL-6 signaling improves the locomotor function in mice with spinal cord injury (Tan et al., 2013). It is therefore plausible to assume that the neuromuscular complications of SARS-CoV-2 infection are due, at least in part, to an increased production of this inflammatory mediator, which is to some extent self-powered by infected MNs.

The expression of genes involved in the apoptotic pathway, such as BCL2, BAX, and CASP8, was upregulated as well. Similar results were recently documented by Li et al. (2020) in SARS-CoV-2 infected lung epithelial cells where an increase of Caspase 8 responsible for cell death and inflammation was observed. Nonetheless, we also observed that the ratio between the anti-apoptotic BCL2 and pro-apoptotic BAX genes was significantly increased in SARS-CoV-2 infected iPSC-MNs, suggesting that programmed cell death is somehow prevented following infection in these neuronal cells. Further analyses will be necessary to verify if this result reflects the virus’ ability to manipulate, delay, or inhibit the host defense response, that usually exploits the apoptotic process to disrupt virus multiplication and propagation, thereby preserving other cells and surrounding tissue.

We also reported a modulation in the expression of factors involved in the antigen processing and presentation pathway. In particular, a statistically significant reduction in the expression of both HLA-A (MHC-I) and ERAP1 was observed. This is not surprising, as modifications in HLA-I and ERAPs expressions are found in many viral diseases. The downregulation of these targets represents one of the immune evasion strategies most widely used by viruses to block antigen presentation as well as CD8 + and NK cell response (Jonjić et al., 2008; Chaudhry et al., 2020; Mancini and Vidal, 2020). SARS-CoV-2 is no exception, as it was documented to downregulate cell surface MHC-I as a strategy to escape immune responses in various peripheral cell models (Zhang et al., 2020; Yoo et al., 2021). The role of MHC-I expression/activation in diseased MNs remains contradictory, as both detrimental and protective effects have been documented (Nardo et al., 2016). Besides immunological mechanisms, MHC-I has been firmly implicated in neuronal plasticity, regulation of synaptic density and axonal regeneration in the CNS and PNS, both during development and in brain diseases. Therefore, the downregulation of MHC-I along with ERAP1 detected in our model of SARS-CoV-2-infected iPSC-MNs might represent: (i) an efficient antagonism of adaptive immune responses favoring successful viral replication, and/or also (ii) a consequence of virus-induced alterations of neuronal homeostasis. This calls for additional focused investigations on the role and functional significance of neuronal MHC-I expression in the frame of SARS-CoV-2 infection.

In line with the possible alterations of neuronal homeostasis associated with SARS-CoV-2 infection in iPSC-MNs, we also detected a consistent downregulation of ANG (Angiogenin) gene expression. ANG is expressed in neurons during neuro-ectodermal differentiation and it exerts both neurotrophic and neuroprotective functions in the light of its role in multiple steps of RNA metabolism or processing (Thiyagarajan et al., 2012). Again, ANG acts as a circulating protein induced during inflammation and exhibits microbicidal activity against bacterial and fungal systemic pathogens, thus contributing to systemic responses to infection (Hooper et al., 2003; Noschka et al., 2021). Our data suggest that SARS-CoV-2 infection in iPSC-MNs goes along with impaired ANG transcription, which might contribute to sustaining both virus-induced immune antagonism and dysregulation of neurotrophic responses.

Finally, in our model of SARS-CoV-2-infected iPSC-MNs, we detected an upregulation of CD147 and S1PR1 (Sphingosine 1-phosphate Receptor 1) genes. CD147 silencing was shown to reduce SARS-CoV-2 replication in epithelial lung cells (Fenizia et al., 2021). Remarkably, CD147 upregulation has been documented in neurons, axons, and capillaries of Alzheimer’s disease brain tissues (Nahalkova et al., 2010). These data configure CD147 as a potential target at the crossroad of SARS-CoV-2 infection and related neurological abnormalities, which remains to be confirmed in other neuronal models.

S1P is a bioactive sphingolipid with pleiotropic functions in many tissues, including the NS, where it regulates neurogenesis and inflammation through the S1P/S1PR axis (Meacci et al., 2020). For instance, accumulation of S1P in the brain promotes glial cell activation, TRL4 upregulation, and IL-6 secretion (Dusaban et al., 2017; O’Sullivan et al., 2018). In SARS-CoV-2 infection the study of the role of S1P at peripheral level has led to contradictory results (Marfia et al., 2021; Torretta et al., 2021). Remarkably, Fingolimod (FTY720), an approved drug for the treatment of multiple sclerosis (MS), which promotes the irreversible internalization and degradation of bound S1PR, has also been tested for COVID-19 treatment, in the light of its potential ability to inhibit the cytokine storm (Meacci et al., 2020). Thus, the observed S1PR1 upregulation might play an important role in SARS-CoV-2 infection in MNs, possibly by fostering pro-inflammatory cytokine release.

Overall, these data are in line with those profiled in other SARS-CoV-2 infected neuronal cell models (Kathuria et al., 2020; Gugliandolo et al., 2021; Samudyata et al., 2021; Valeri et al., 2021), suggesting an embedded mess-up of the transcriptional machinery affecting the cellular equilibrium. Notwithstanding, it appears rather difficult to pinpoint a shared and precise expression profile fostered by SARS-CoV-2 infection in this motor neuronal cell model.

The aforementioned lack of the immune system, a certain level of heterogeneity in cell differentiation, and the restricted number of enrolled subjects represent limiting factors in the interpretation of the results. Last but not least, the lack of an *in vivo* blood-CNS barrier represents a major inherent limitation of the present model. However, we believe that the present results contribute to adding to previous evidence that supports the ability of SARS-CoV-2 to affect different neuronal populations after entering the CNS parenchyma via direct or indirect BBB impairment (Pellegrini et al., 2020; Song et al., 2021; Lyoo et al., 2022; Stein et al., 2022; Kettunen et al., 2023). Indeed, our data are the first to document the susceptibility of iPSC-MNs to productive SARS-CoV-2 infection and the ensuing alteration of their homeostasis. Yet, the neuroinvasive routes of SARS-CoV-2 entry within the gray matter of the spinal cord specifically, remain to be investigated by employing *in vivo* models. Remarkably, during the revision of the present paper, a preprint was published on bioRxiv, documenting that SARS-CoV-2 can productively infect spinal cord neurons in mice models (Joyce et al., 2022). The study suggest that SARS-CoV-2 infection of spinal cord motor neurons might occur following virus spread from brainstem neurons. It also detected productive infection in sensory ganglionic neurons of the PNS (Joyce et al., 2022), suggesting the existence of an alternative, retrograde axonal pathway that fosters *trans*-synaptic viral spread from periphery to sensory ganglionic neurons, interneurons and motor neurons. Coupled with our results, these data suggest that SARS-CoV-2 is able to establish productive infection in unassessed nervous system sites, potentially causing sensory and neuromuscular symptoms associated with COVID-19 (Jacob et al., 2022). Further research is needed to understand how changes in motor neurons might contribute to the acute and long term neurologic sequelae. These findings could help uncover the biological basis of neuromuscular disorders and identify new therapeutic targets to counteract the neurological symptomatology.

## Consent for publication

All authors give consent for the publication of the manuscript.

## Data availability statement

The original contributions presented in the study are included in the article/Supplementary material, further inquiries can be directed to the corresponding author.

## Ethics statement

The studies involving humans were approved by the Local Ethics Committee (approval number 2022_03_15_12). The studies were conducted in accordance with the local legislation and institutional requirements. The participants provided their written informed consent to participate in this study.

## Author contributions

GC: Formal analysis, Investigation, Methodology, Writing – original draft. CC: Formal analysis, Investigation, Methodology, Writing – original draft. FL: Formal analysis, Methodology, Writing – original draft, Conceptualization, Data curation. SI: Methodology, Data curation, Writing – original draft. MG: Methodology, Writing – original draft, Formal analysis, Investigation. CV: Formal analysis, Investigation, Writing – original draft, Data curation. CM: Investigation, Writing – original draft, Methodology. SS: Investigation, Writing – original draft, Data curation. SZ: Data curation, Investigation, Writing – original draft, Methodology. DT: Writing – original draft, Conceptualization. VS: Funding acquisition, Writing – review and editing. MC: Funding acquisition, Writing – review and editing. AR: Writing – review and editing. MB: Writing – review and editing, Conceptualization, Supervision.

## Footnotes

1
www.cdc.gov

2
http://www.proteinatlas.org
