# Supplementary material for: Human motor neurons derived from induced pluripotent stem cells are susceptible to SARS-CoV-2 infection
Source: Front Cell Neurosci. 2023 Dec 5;17:1285836. doi: 10.3389/fncel.2023.1285836 (PMC10728732; doi:10.3389/fncel.2023.1285836)

Supplementary Table S1

Table S1 Number of independent replicates performed for each assay with the iPSC-MN lines obtained from 3 healthy control subjects already described in PMID: 31382054 (Ref.48) (S1 and S2) and in PMID: 29800782 (Ref. 49) (S3). Features of the enrolled subjects (sex, age) are also reported.

	Sex	Age	Real-time PCR for N1 and N2 viral genes	TCID50 on VERO E6 cells	Gene expression analysis by Real-Time PCR	Quantigene assay	IF	Trypan Blue exclusion assay
S1	M	37	n=5	n=1	n=5	n=2	n=5	n=5
S2	F	45	n=5	n=1	n=5	-	n=4	n=5
S3	F	49	n=4	n=1	n=4	n=1	n=4	n=4

S= subject

Supplementary Table S2

Table S2. Scheme of target genes analysed by Real-time PCR array in SARS-CoV-2 infected iPSC-MNs. *ACTB* and *GAPDH* were used as housekeeping genes for data normalization

**ACE2**	**CD147**	**ERK**	**HLA-A**	**MMP9**	**TGFB1**
**ACTB**	**C5AR1**	**EPHA4**	**IFITM1**	**MT2A**	**TIMP1**
**ANG**	**CLDN5**	**FOS**	**IFITM3**	**MX1**	**TLR1**
**ANXA2**	**CTSB**	**FURIN**	**IGF1**	**NANOS2**	**TLR2**
**BAX**	**CXCR4**	**GAPDH**	**IL1A**	**NFKB**	**TLR4**
**BCL2**	**CYFIP2**	**GRN**	**IL6**	**NRP1**	**TLR6**
**BDNF**	**ERAP1**	**GSK3B**	**IL8**	**S100B**	**TLR8**
**CASP8**	**ERAP2**	**HDAC1**	**JNK**	**S1PR1**	**VEGFA**

Supplementary Table S3

Table S3. Cell viability assessment in mock and SARS-CoV-2 infected iPSC-MNs

	Samples		
	**S1**	**S2**	**S3**
Mock x	93%	93%	96%
Mock y	93%	93%	95%
Mock z	93%	91%	96%
**mean**	**93%**	**92%**	**96%**
Infected x	93%	93%	92%
Infected y	94%	94%	96%
Infected z	95%	93%	92%
**mean**	**94%**	**93%**	**93%**

S= subject

Fig. S1. Expression of SARS-CoV-2 human receptors on iPSC-MNs and A549-hACE2 cells. Quantification of Fluorescence intensity of ACE2, CD147, NRP1 in A549-hACE2 cells and iPSC- MNs obtained by Immunofluorescence assay. Results are presented as mean ± SEM; n ≥ 3 for each cell line/iPSC-MN; the *Student's t-test* was used with the *p* value threshold of 0.05. Significance is indicated as follows: *p<0.05.

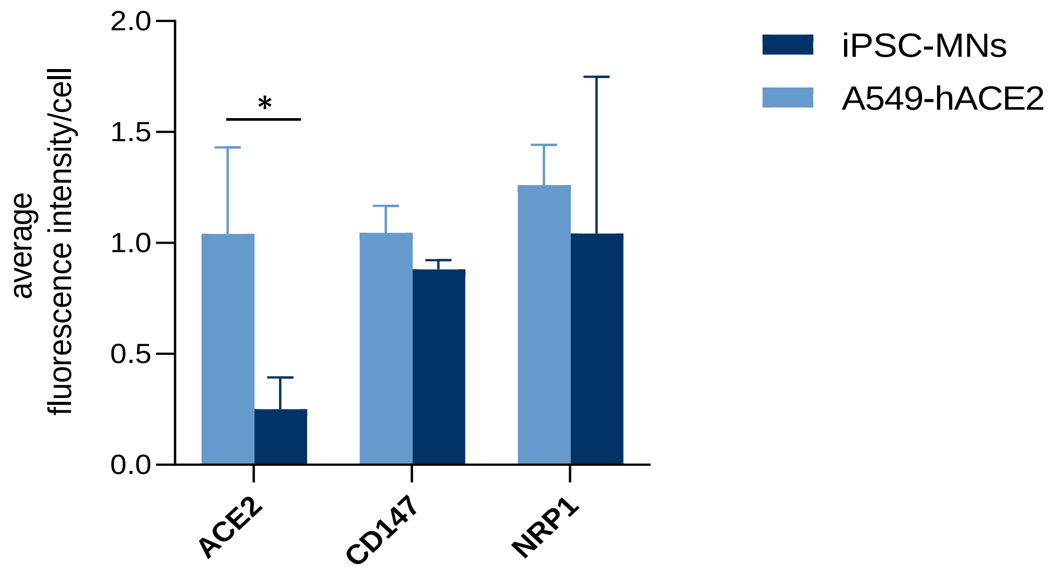

Fig S2. Assessment of viral replication in Vero E6 cells. Upon *in vitro* challenge of iPSC-MNs with 1 MOI of SARS-CoV-2 the infection was monitored at 24hpi, 48hpi and 72hpi. Results correspond to the absolute viral copy number/µl of the SARS-CoV-2 N1 and N2 target sequences from cell supernatants that were quantified through a single-step Real-time qPCR by referring to a standard curve for Ct values (IDT, Coralville, IA, USA). Results are presented as mean ± SEM from ≥3 independent replicates.

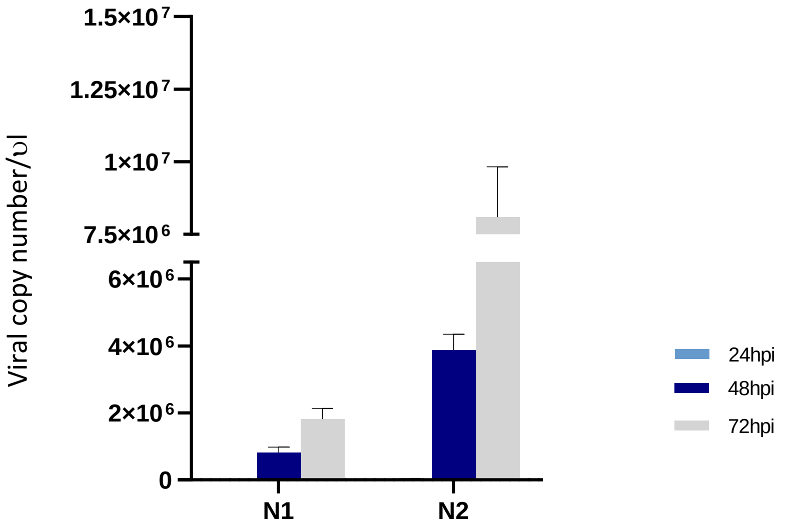

Fig S3. BCL2/BAX mRNA expression ratio. The ratio between the anti-apoptotic BCL2 and the pro-apoptotic BAX mRNA expression significantly increased in SARS-CoV-2 infected compared to uninfected (Mock) iPSC-MNs at 72hpi. Results are presented as mean ± SEM from ≥4 independent replicates on iPSC-MNs derived from the 3 enrolled subjects. Significance is indicated as follows: * = $p < 0.05$.

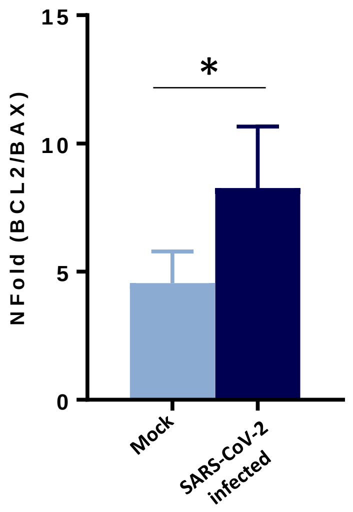

Supplement: Supplementary file 1 [file Data_Sheet_1.pdf]
